# **Role of innate immune and inflammatory signaling in West Nile virus tropism and neuronal and glial cell death**

**DOI:** 10.1038/s41598-025-27954-2

**Published:** 2025-12-19

**Authors:** Valentine Chaillot, François Piumi, Kamila Gorna, Noémie Berry, Jennifer Richardson, Alexandra Benchoua, Muriel Coulpier

**Affiliations:** 1 UMR Virologie, Anses, INRAE, École Nationale Vétérinaire d’Alfort, Université Paris-Est Créteil, Maisons-Alfort, France; 2 Université Paris-Saclay, Gif-Sur-Yvette, France; 3 CECS, I-STEM, AFM, Corbeil-Essonne, France

**Keywords:** Neurotropic virus, Neuropathogenesis, Innate immune response, Inflammatory response, Interferon-stimulated genes, IFI6, Immunology, Neuroscience

## Abstract

West Nile virus (WNV) is a mosquito-borne virus that causes severe neurological disease in humans. Despite substantial advances, our knowledge of the mechanisms involved in damaging the human brain is still limited. To address this gap, we developed a physiologically relevant in vitro model using human neuronal/glial cells and aimed to determine WNV tropism, assess whether the virus induces innate immune and inflammatory responses, and elucidate the resulting pathophysiological consequences. We found that WNV productively infected glial cells, whereas neurons exhibited a remarkable and unexpected resistance to infection. Despite the induction of a robust innate immune response mediated by IFN signalling and a rapid control of WNV replication in glial cells, we observed substantial death of astrocytes, oligodendrocytes, and neurons. Analysis of cytokine and chemokine expression further revealed that infection triggered an inflammatory response, potentially contributing to bystander cell death. We also showed that IFN signaling did not contribute to the resistance of neurons and identified IFI6 as an effector of the antiviral response in human glial cells. Together, our results underscore the importance of human neural models for confirming previous findings obtained in less physiologically relevant models and for unravelling novel cellular and molecular mechanisms.

**Supplementary Information:**

The online version contains supplementary material available at 10.1038/s41598-025-27954-2.

## Introduction

West Nile virus (WNV) is a zoonotic, mosquito-borne pathogen belonging to the *Orthoflavivirus* genus of the *Flaviviridae* family. WNV can cause severe, sometimes fatal, neurological disease in humans and horses^1^. Over the past two decades, WNV has re-emerged as a significant global public health concern, driven by its expanding geographical distribution and the growing number of outbreaks, especially in Europe and North America^2^. Now endemic on every continent except Antarctica, WNV causes an estimated 2500 and 1500 human cases annually in the United States of America and the European Union, respectively^3,4^. While the majority of human infections remain asymptomatic, clinical cases can present as mild flu-like symptoms, such as fever, headache and fatigue. In fewer than 1% of cases, however, the virus invades the central nervous system (CNS), leading to a neuroinvasive disease manifesting as encephalitis, meningitis and/or acute flaccid paralysis, with fatality rates ranging from 10 to 30%^5^. Despite the high morbidity and mortality associated with neurological WNV infection, no vaccine or specific antiviral treatment is currently available for human use^6^.

WNV is primarily transmitted to humans through the bite of infected mosquitoes, predominantly of the *Culex* family, although alternative transmission routes such as blood transfusion or organ transplantation have also been reported^7^. Following inoculation into the skin, WNV initially replicates in keratinocytes and Langerhans cells. The latter infected cells are thought to migrate to the local lymph nodes where the virus replicates further, particularly in leukocytes, before entering the bloodstream and disseminating to peripheral organs^8^. At this stage, the virus may in some cases breach the CNS, though the precise mechanisms remain incompletely understood. Beyond crossing the blood–brain barrier, the virus may also invade the brain via the transneural route, traveling along the axons of peripheral nerves^9^. Once within the CNS, WNV causes characteristic neuropathological lesions, including perivascular lymphocytic infiltrates, microglial nodules, astrogliosis and loss of neurons^10–12^. As WNV antigens are predominantly detected in neurons in vivo and severe neuronal loss is observed, neurons are considered the principal targets of WNV infection in the CNS^11–13^. Resident glial cells, particularly astrocytes and microglia, also play key roles in WNV neuropathogenesis through the release of pro-inflammatory cytokines and chemokines. While these factors are essential for controlling viral replication, they can also exert cytotoxic effects or promote the infiltration of peripheral immune cells, exacerbating neuro-inflammation and cell damage^13,14^. Nevertheless, the extent to which viral infection of glial cells contributes to neuropathology has not been fully investigated in human brain cells.

The cellular and molecular mechanisms underlying WNV-induced neuropathogenesis have been studied primarily in vivo using rodent models, or in vitro using immortalized cell lines, sometimes lacking a neural phenotype, or primary murine neural cells, which are more readily available than those of human origin^15^. Unfortunately, findings from these studies can be difficult to extrapolate to human neuropathogenesis, due to cell-type and interspecies differences in certain cellular pathways, especially in those involved in the innate immune response^16^. There is thus growing recognition of the importance of using models based on human CNS cells to study neurotropic human viral infections. Although primary human neural cells are occasionally used, their limited availability restricts broader application. In contrast, neural cells derived from fetal neural progenitors, embryonic stem cells or induced pluripotent stem cells offer renewable, on-demand sources and have become valuable tools for investigating virus-host interactions in the CNS^17^. While these cells are often differentiated into a single cell type, models incorporating multiple neural cell types are increasingly being employed to reflect the cellular complexity of the CNS more faithfully. Such multiple cell culture systems have already proved useful in virology in the study of cellular interactions occurring during infection^18–20^.

In this study, we infected neuronal/glial cells derived from human fetal neural progenitors with WNV to establish a physiologically relevant in vitro model and to investigate the relationship between WNV tropism, innate and inflammatory responses and cell damage. To our knowledge, this is the first study to address all these aspects in a complex culture model of primary-like human brain cells.

## Materials and methods

### Cell culture

Human neural progenitor cells (hNPC) were prepared and cultured as described in Brnic et al*.* (2012)^21^, and differentiated into human neuronal and glial cells (hNGC) as described in Fares et al*.* (2020)^20^. Briefly, hNPC were seeded at a density of 44,000 cells/cm^2^ in culture plates coated with Matrigel™ (#354,230, Corning, USA). Differentiation into a mixed population of neuronal and glial cells was induced 24 h after plating by replacing N2A medium with 1:1 N2A and NBC media and withdrawing Epidermal Growth Factor (EGF, #PCYT-217, Eurobio Scientific, France) and basic Fibroblast Growth Factor (bFGF, #PCYT-218, Eurobio Scientific, France). N2A is composed of Advanced Dulbecco’s modified Eagle medium-F12 (#12,634,028, Gibco, Thermo Fisher Scientific, USA) supplemented with 2 mM L-glutamine (#25,030,081, Gibco, Thermo Fisher Scientific, USA), 0.1 mg/ml apo-transferrin (#T1147, Sigma-Aldrich, USA), 25 μg/ml insulin (#I9278, Sigma-Aldrich, USA), and 6.3 ng/ml progesterone (#P6149, Sigma-Aldrich, USA). NBC is composed of neurobasal medium (#21,103,049, Gibco, Thermo Fisher Scientific, USA) supplemented with 2 mM L-glutamine and B27 without vitamin A 1X (#12,587,010, Gibco, Thermo Fisher Scientific, USA). Differentiation conditions were maintained for 13 days with medium replacement twice a week, prior to infection. Ninety-six-well plates (#655,090, Greiner Bio-One, Austria) were used for fluorescent immunostaining, and 24-well plates (#353,047, Falcon, Corning, USA) were used to prepare lysates for RNA analysis.

Late cortical progenitor-like (LCP) cells were obtained from human induced pluripotent stem cells and differentiated into cortical glutamatergic neurons as described in Boissart et al*.* (2013)^22^. LCP were seeded at a density of 35,000 cells/cm^2^ in 384-well plates and differentiation conditions were maintained for 28 days.

VERO E6 (ATCC No. CRL-1586) cells were cultured in Dulbecco’s modified Eagle medium (#61,965,026, Gibco, Thermo Fisher Scientific, USA) supplemented with 10% fetal bovine serum (FBS, #CVFSVF00-01, Eurobio Scientific, France), 1% sodium pyruvate (#11,360,070, Gibco, Thermo Fisher Scientific, USA) and 1% penicillin–streptomycin (#15,140,122, Gibco, Thermo Fisher Scientific, USA).

### Ethics approval and consent to participate

Human fetus was obtained after legal abortion with written informed consent from the patient. The procedure for the procurement and use of human fetal central nervous system tissue was approved and monitored by the “Comité Consultatif de Protection des Personnes dans la Recherche Biomédicale” of Henri Mondor Hospital, France. All methods were in compliance with relevant French laws and institutional guidelines. Authorization and declaration numbers from the French Research Ministry are AC-2017–2993 (CHU Angers) and DC-2019–3771 (UMR Virologie). The rabbit immunization protocol (anti-WNV-E3 antibody) complied with EU legislation (authorization 12/04/11–6 accorded by the ANSES/ENVA/UPEC ethical committee).

### Virus and infection

Three different WNV strains were used: WNV_NY99_ (an American strain of lineage 1, Genbank Accession No. KC407666.1), WNV_FR2015_ (a European strain of lineage 1, Genbank Accession No. MT863559.1) and WNV_FR2018_ (a European strain of lineage 2, Genbank Accession No. MT863561.1). The three strains were kindly provided by Dr. Gaëlle Gonzalez (ANSES, Maisons-Alfort, France). Working stocks (Passage 4) were generated in VERO cells (VERO-ATCC-CCL81) cultured in DMEM medium, supplemented with 2% FBS. WNV_FR2015_ and WNV_FR2018_ were propagated once (at passage 2) in C6/36 cells. Titers were estimated by plaque assay on VERO cells as described in Donadieu et al. (2013)^23^.

HNGCs differentiated for 13 days were infected at the indicated MOI or treated with culture medium only (“Mock”) for 90 min at 37 °C before removal of the inoculum. Subsequently, the cells were washed with 100 µL/well of fresh N2A/NBC medium. Immediately afterward, 60 µL/well was collected (called “wash”) and replaced by fresh medium until collection of supernatants and/or cell lysates at the indicated time points. Virus titers were estimated by endpoint dilution on VERO cells (TCID50), following the Reed and Muench method^24^. All procedures involving infectious materials were performed under bio-safety level-3 conditions.

### Immunofluorescence assay

HNGC were fixed for 30 min in 4% paraformaldehyde (#15710, Electron Microscopy Sciences, USA) in PBS 1X and standard immunofluorescence was performed using antibodies for HuC/HuD (1:500, mouse, #A21271, Thermo Fisher Scientific, USA), βIII-tubulin (1:1000, mouse, #T8660, Sigma-Aldrich, USA or 1:1000, rabbit, #ab18207, Abcam, UK), Glial Fibrillary Acidic Protein (GFAP, 1:1000, mouse, #G3893, Sigma-Aldrich, USA or 1:1000, rabbit, Z0334, Dako, Denmark), Oligodendrocyte transcription factor 2 (OLIG2, 1:1000, goat, #AF2418, R&D Systems), cleaved caspase-3 (1:100, rabbit, #9661, Cell Signaling Technology, USA), S100β (1:1000, rabbit, #ab52642, Abcam, UK), double-stranded RNA (dsRNA, 1:800, mouse, #10020200, Scicons, Hungary) and the domain 3 of WNV envelope protein (WNV-E3, 1:1000, rabbit, in house). Cells were blocked for 2 h in 3% BSA (#A9647, Sigma-Aldrich, USA), 0.3% Triton-X-100 (VWR Chemical, Belgium) in PBS 1X. Primary antibodies were diluted in 0.3% BSA, 0.03% Triton-X-100 in PBS 1X, and incubated overnight at 4 °C. Secondary antibodies were Alexa Fluor-488/546/594-conjugated anti-mouse/anti-rabbit/anti-goat IgG (Molecular Probes, Invitrogen, Thermo Fisher Scientific, USA), diluted at 1:1000 and incubated for 2 h at room temperature. Nuclei were stained with 4′,6-diamidino-2-phenylindole (DAPI) (Life Technologies, Thermo Fisher Scientific, USA) at 0.1 ng/ml.

### Image acquisition and analysis

The digitalized images shown were acquired with an AxioObserver Z1 (Zeiss, Germany) inverted microscope using ZEN software (v3.5, Zeiss, Germany) and were adjusted for brightness and contrast using this software.

To enumerate infected cells, three channel images were acquired in a fully automated and unbiased manner using the Opera Phenix™ Plus High-Content Screening System (Revvity, USA) and a 10 × air objective (NA = 0.3). Twelve images per channel per well (representing approximately 85% of the entire well) were acquired and analyzed with Signals Images Artist Analysis and Management software (SImA, Revvity, USA), using a customized algorithm for cell segmentation and identification. Briefly, nuclei were segmented based on DAPI staining. Living and dead cells were distinguished by the mean nuclear intensity, with dead cells exhibiting higher DAPI signal. Infected cells were enumerated by quantifying the intensity of WNV-E3 immunostaining in a perinuclear ring surrounding living nuclei. The threshold for WNV-E3 positivity was set at the lowest intensity value that reliably distinguished perinuclear WNV-E3 signal from background staining. Astrocytes were identified by the size of their nuclei (larger than those of neurons). Oligodendrocytes, were identified by immunostaining for OLIG2 in the nuclear region. Total infection refers to the percentage of astrocytes and oligodendrocytes infected relative to the total cell population. Astrocyte infection and oligodendrocyte infection refer to the percentage of infected cells within the astrocyte or oligodendrocyte populations, respectively.

For automated quantification of cells immunostained with antibodies directed against HuC/HuD and OLIG2 and of cell processes immunostained with antibodies against βIII-tubulin and GFAP, images were acquired using the ImageXpress micro automated microscope (Molecular Devices, UK) and analyzed using Custom Modules designed using MetaXpress Analysis Software V6).

### Semi-quantitative quantification of cytokines in cell supernatant

The Proteome Profiler Human Cytokine Array kit (#ARY005B, R&D Systems, USA) was used to assess the impact of WNV infection on cytokine secretion in hNGC. It was used following the manufacturer’s instructions. Briefly, hNGC were cultured on 24-well plates and infected with WNV_NY99_ (MOI 10) for 24 h. Collected supernatants were pooled from three wells for each condition (500 µL/well) and were inactivated by UV-irradiation (254 nm, 2 J/cm^2^), using a CL-508 Crosslinker (Uvitec, UK). Inactivated supernatants (700 µL) were mixed with array buffers and the “Human Cytokine Array Detection Antibody Cocktail” before being incubated overnight at 4 °C with pre-blocked membranes spotted in duplicate with 36 antibodies for a variety of cytokines and chemokines (Supplementary table 1). Streptavidin-HRP was prepared at 1:2000 dilution in array buffer and added to the membranes for 30 min at room temperature. The array “Chemi Reagent Mix” was distributed evenly on each membrane before visualization with the ChemiDoc MP imaging system (Bio-Rad Laboratories, USA). Relative quantification was performed using ImageJ (v1.54 g) software by measuring the inverted grayscale intensity of each individual spot and normalizing it to the mean intensity of the designated reference spots.

### Induction or inhibition of antiviral response in hNGC

To assess the impact of the IFN signaling pathway on WNV infection in hNGC, cells were pretreated with recombinant human IFN-β (100 U/mL, #11410–2, PBL Assay Science, USA) or with ruxolitinib (5 µM, #S1379, Selleck Chemicals LLC, USA), a JAK 1/2 inhibitor, for 2 h before infection with WNV (MOI 10). After removal of the inoculum, a fresh IFN-β or ruxolitinib dilution was added. At 24 h post-infection or treatment, cells were fixed or lysed and supernatants were harvested for subsequent analysis.

### IFI6 downregulation

SiRNA targeting IFI6 was purchased from Horizon Discovery (Si-genome in SMARTpool format, #M-003672–02-0005). Human NGC cultured in 96-well plates were transfected with 25 nM of siRNA and 0.2 μL of DharmaFECT 1 Transfection reagent (Horizon Discovery, UK) as per manufacturer’s instructions. Forty-eight hours after transfection, RNAi-transfected cells were infected with WNV_NY99_ at an MOI of 10. Viral inoculum was removed 90 min later and replaced with 150 μL of fresh N2A/NBC medium. Cells were fixed and supernatants were collected 24 h post-infection. The impact of RNAi on viral infection was assessed by immunofluorescence labeling of infected cells and by quantification of WNV genomic RNA in supernatants by reverse transcriptase quantitative polymerase chain reaction (RT-qPCR).

### RNA isolation and RT-qPCR

RNA was isolated from infected and non-infected hNGC. Cells were lysed and RNA extracted using the RNEasy mini kit (#74106, Qiagen, Germany), following the manufacturer’s instructions. Extraction of viral RNA from supernatants of infected cells was performed using QIAamp Viral RNA Mini Kit (#52904, Qiagen, Germany), according to the manufacturer’s instructions. One hundred nanograms of RNA from cell lysates and 2 μL of RNA from supernatant were used for cDNA synthesis using the SuperScript™ II Reverse Transcriptase kit (#18064022, Thermo Fisher Scientific, USA). Real-time PCR was performed in a total reaction volume of 10 µL, using 2 μL of cDNA and QuantiTect SYBR Green PCR master mix (Qiagen, Germany), on a LightCycler™ 96 instrument (Roche Applied Science, Germany). Samples were held for 15 min at 95 °C and then subjected to 40 amplification cycles consisting of incubations at 95 °C for 30 s, 60 °C for 30 s, and 72 °C for 30 s. This was followed by a final step for melting curve analysis consisting of incubations at 95 °C for 10 s, 58 °C for 60 s, 96 °C for 1 s and 40 °C for 30 s. For relative quantification, the − 2ΔΔCt method was used^25^. GAPDH was used as the reference gene. Primers pairs are listed in Supplemental table 2.

### Statistical analysis

Statistical analyses were performed using GraphPad Prism V10.0.0. Data normality was assessed using the Shapiro–Wilk test. Depending on the distribution and the experimental design, comparisons between two groups were performed using either an unpaired Student’s t test or a Mann–Whitney test. For comparisons between multiple time points, a one-way ANOVA analysis followed by a Tukey’s test or a Kruskal–Wallis test followed by a Dunn’s test was used. Statistical tests applied are specified in the legend of each figure.

## Results

### Human brain cells differentiated from fetal neural progenitors are susceptible to WNV but control infection

**Fig. 1:**
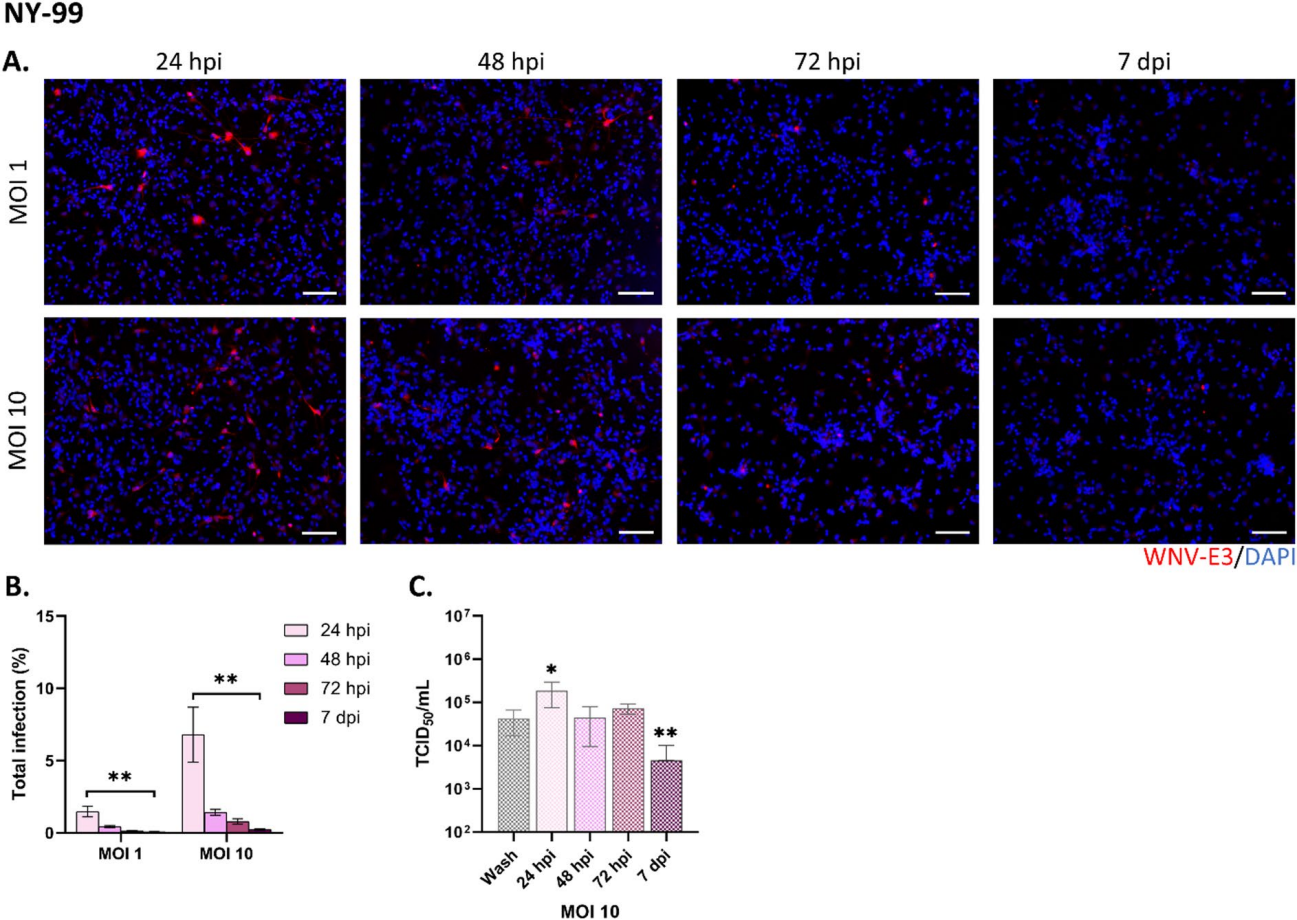
Permissivity of neuronal/glial cells derived from human neural progenitors to WNV_NY99_. (**A**) Immunofluorescence labeling with an antibody against WNV-E3 (red) of hNGC infected for 24, 48, 72 h or 7 days with WNV_NY99_ at MOI 1 or 10. Nuclei are stained with DAPI (blue). Scale bars = 100 µm, (**B**) Automatic enumeration of infected cells based on immunofluorescence staining using an OPERA-Phenix™ Plus instrument, (**C**) Supernatant of WNV_NY99_-infected hNGC (MOI 10) were titrated (TCID50) at the indicated time points. Results are representative of three independent experiments performed in six replicates (**B**) or pooled from two independent experiments performed in at least two replicates (**C**). Data are expressed as the mean ± SD. Statistical analysis was performed using a Kruskal–Wallis test with post-hoc Dunn’s test (**B**) or two-tailed unpaired Mann–Whitney tests between the wash and other timepoints (**C**) with GraphPad Prism V10.0.0. *p < 0.05; **p < 0.01.

To characterize human brain cell infection by WNV, we used conditions similar to those previously described in Fares et al*.* (2020)^20^. Human neural progenitor cells (hNPC) of fetal origin were differentiated for 13 days into neuronal/glial cells (hNGC) — at which time all cells were shown to be differentiated and quiescent^26^ — before infection with WNV. We examined the capacity of the WNV_NY99_ strain to infect, replicate and disseminate in hNGC at MOI 1 and 10, from 24 h post-infection (hpi) to 7 days post-infection (dpi). Immunostaining of WNV-infected hNGC with an antibody directed against the domain 3 of the WNV envelope protein (WNV-E3) revealed that at high MOIs (1, 10) the virus infected human brain cells, as observed at 24 hpi, but did not disseminate within the neuronal/glial culture at later time points (Fig. 1A). Instead, the percentage of infected cells decreased over time from 48 hpi to 7 dpi, as shown by enumeration of infected cells (Fig. 1B). Indeed, whereas 6.8 ± 0.4% of cells were infected at 24 hpi (MOI 10), this number rapidly dropped to 1.4 ± 0.2%, 0.8 ± 0.2% and 0.2 ± 0.1% at 48 hpi, 72 hpi and 7 dpi, respectively. This was confirmed by quantification of viral titer by endpoint dilution, which showed an increase in infectious viral particles in cell supernatant at 24 h after infection, revealing productive infection, followed by a decrease at 48 hpi, 72 hpi and 7 dpi (Fig. 1C). In order to verify whether this pattern of infection was specific to WNV_NY99_ or, on the contrary, could be generalized to other WNV strains, we reproduced the experiment using WNV_Fr2015_ and WNV_Fr2018_, two European strains of lineage 1 and lineage 2, respectively. For both viruses, the results obtained were similar to those observed with WNV_NY99_, albeit with slightly higher percentages of infected cells and viral titers at 24 hpi for WNV_Fr2015_ and WNV_Fr2018_ than for WNV_NY99_ (Supplemental Fig. 1A–F). Thus, our results showed that despite an initial productive infection of WNV in hNGC, it was strongly and rapidly controlled, leading to a marked decrease in infection.

### WNV infects human astrocytes and oligodendrocytes but not human neurons

**Fig. 2:**
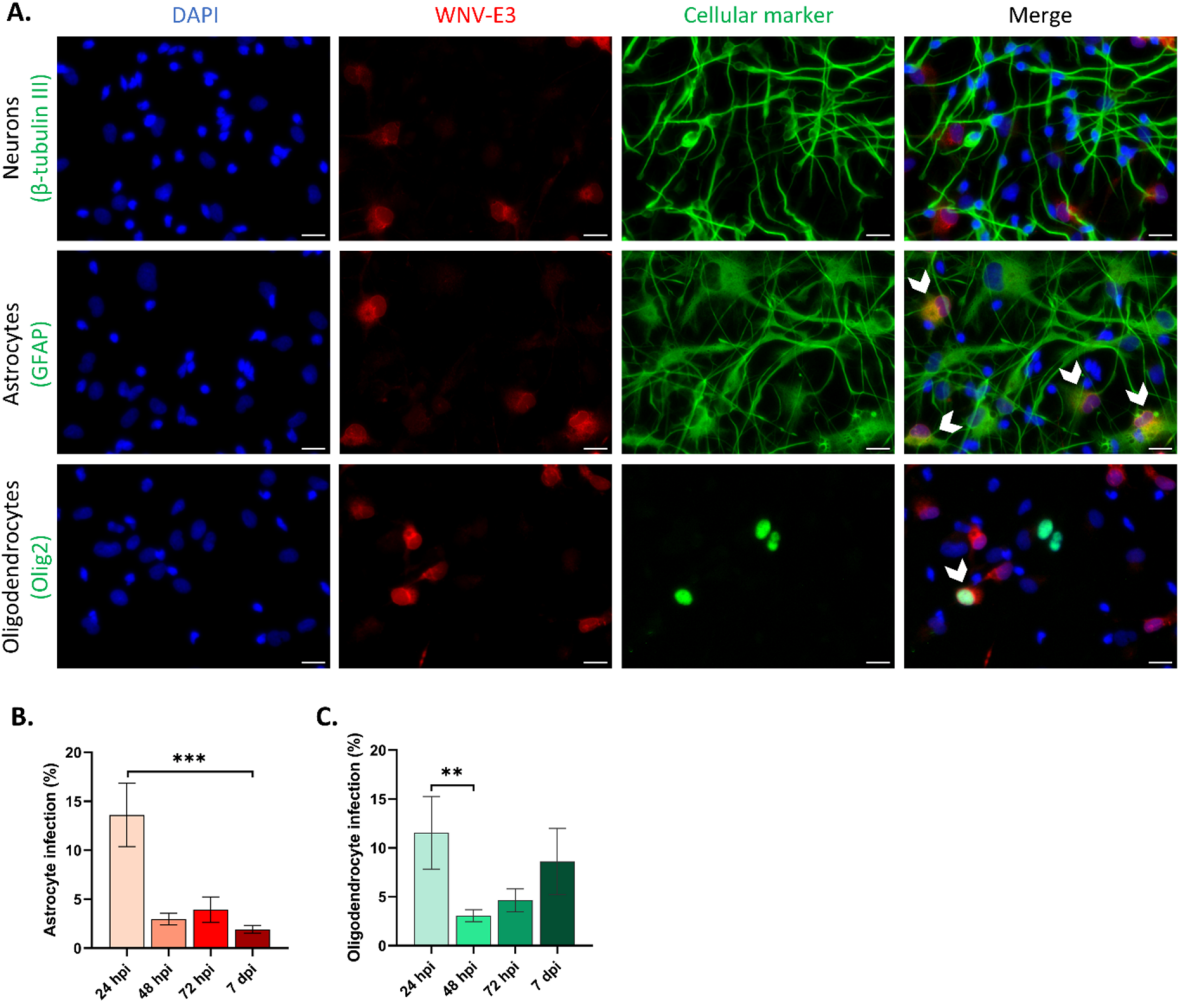
WNV_NY99_ tropism in hNGC. hNGC were infected with WNV_NY99_ at MOI 10 for 24, 48, 72 h and 7 days. (**A**) Immunofluorescence labeling of infected cells at 24 hpi. Antibodies against βIII-tubulin (neurons), GFAP (astrocytes) or OLIG2 (oligodendrocytes) (green) and WNV-E3 (red) were used. Nuclei were stained with DAPI (blue). Arrowheads show infected astrocytes and oligodendrocytes. Note the absence of infected neurons. Scale bars = 20 µm, (**B**, **C**) Automatic enumeration of infected astrocytes (**B**) or oligodendrocytes (**C**) at 24, 48, 72 hpi and 7 dpi, based on immunofluorescence staining using an OPERA-Phenix™ Plus instrument. Results are representative of three independent experiments performed in at least 5 replicates. Data are expressed as mean ± SD. Statistical analysis was performed using a Kruskal–Wallis test with post-hoc Dunn’s test with GraphPad Prism V10.0.0. **p < 0.01; ***p < 0.001.

We had previously characterized the HNPC-derived hNGC at 13 and 21 days after the onset of differentiation^20,26^. Enumeration of cells based on immunostaining with antibodies directed against HuC/HuD (neuronal marker), GFAP (astrocytic marker) and OLIG2 (oligodendrocyte marker) had shown that the three cellular phenotypes were acquired by day 13 of differentiation and remained stable for up to day 21 of differentiation and that the culture was composed of approximately 70% neurons, 20–30% astrocytes and 5% oligodendrocytes, as determined by automated and manual cell counts^20^. To gain in precision, we performed co-immunostaining with neuronal, astrocytic and oligodendrocytic markers and showed that each nucleus was associated with one of these markers (Supplemental Fig. 2A), indicating that no additional cellular types were present in the culture – in particular microglia, which can infiltrate the fetal brain as early as 3 weeks post-conception^27^. In addition, as GFAP can also be expressed in some radial glial cells^28^, we further validated the astrocytic identity of GFAP-positive cells by co-immunostaining them with S100β, another marker of astrocytes. All GFAP-positive cells were also S100β-positive, confirming their astrocytic phenotype (Supplemental Fig. 2B). Next, in order to determine which cell types are permissive to WNV_NY99_, hNGC infected for durations ranging from 24 h to 7 days were co-immunostained with antibodies specific to neurons, astrocytes or oligodendrocytes along with the WNV-E3 antibody. Strikingly, although WNV has been described as primarily infecting neurons, they remained uninfected in hNGC, at all of the time points we examined. Despite a high proportion of neurons in the cultures, only extremely scarce cells exhibited βIII-tubulin/WNV-Env-E3 co-immunostaining, suggesting that neurons were, in their vast majority, highly resistant to WNV infection (Fig. 2A). On the contrary, GFAP/Env-E3 and OLIG2/Env-E3 co-immunostaining revealed that both astrocytes and oligodendrocytes were permissive to WNV (Fig. 2A). To confirm this glial-specific tropism, we co-immunostained hNGC for double-stranded RNA (dsRNA), a marker of active viral replication, together with cell-type markers. This analysis revealed that WNV replication occurred in astrocytes and oligodendrocytes, but only extremely rarely in neurons (Supplemental Fig. 3A–B), confirming neuronal resistance to WNV infection. Enumeration of both infected astrocytes and oligodendrocytes was performed throughout the course of infection, in order to characterize the virus’s behavior in these two cell types (Fig. 2B, C). The general profile was similar in both cases, showing a peak of infection at 24 hpi followed by a rapid and strong decrease from 48 hpi onward. The level of infection was observed to be similar in the two cell types, with approximately 13.6 ± 3.2% of astrocytes and 11.5 ± 3.7% of oligodendrocytes being infected at 24 hpi. Again, we reproduced the same experiment with WNV_Fr2015_ and WNV_Fr2018_ strains to determine whether different WNV strains may have had distinct tropism for human brain cells (Supplemental Fig. 3C–H). A similar pattern of infection was, however, observed for the three strains, providing no evidence of strain dependency. Finally, we sought to determine whether WNV could infect cortical neurons derived from hiPSC. Co-immunostaining with anti-WNV-E3 antibody and βIII-tubulin at 24 hpi revealed, again, almost no infected neurons (Supplemental Fig. 2I). Thus, human neurons in both hNPC- and hiPSC-derived cultures were highly resistant to WNV infection, and, while human astrocytes and oligodendrocytes were susceptible, viral spreading in these cells did not occur.

### WNV induces the death of glial cells and neurons

**Fig. 3:**
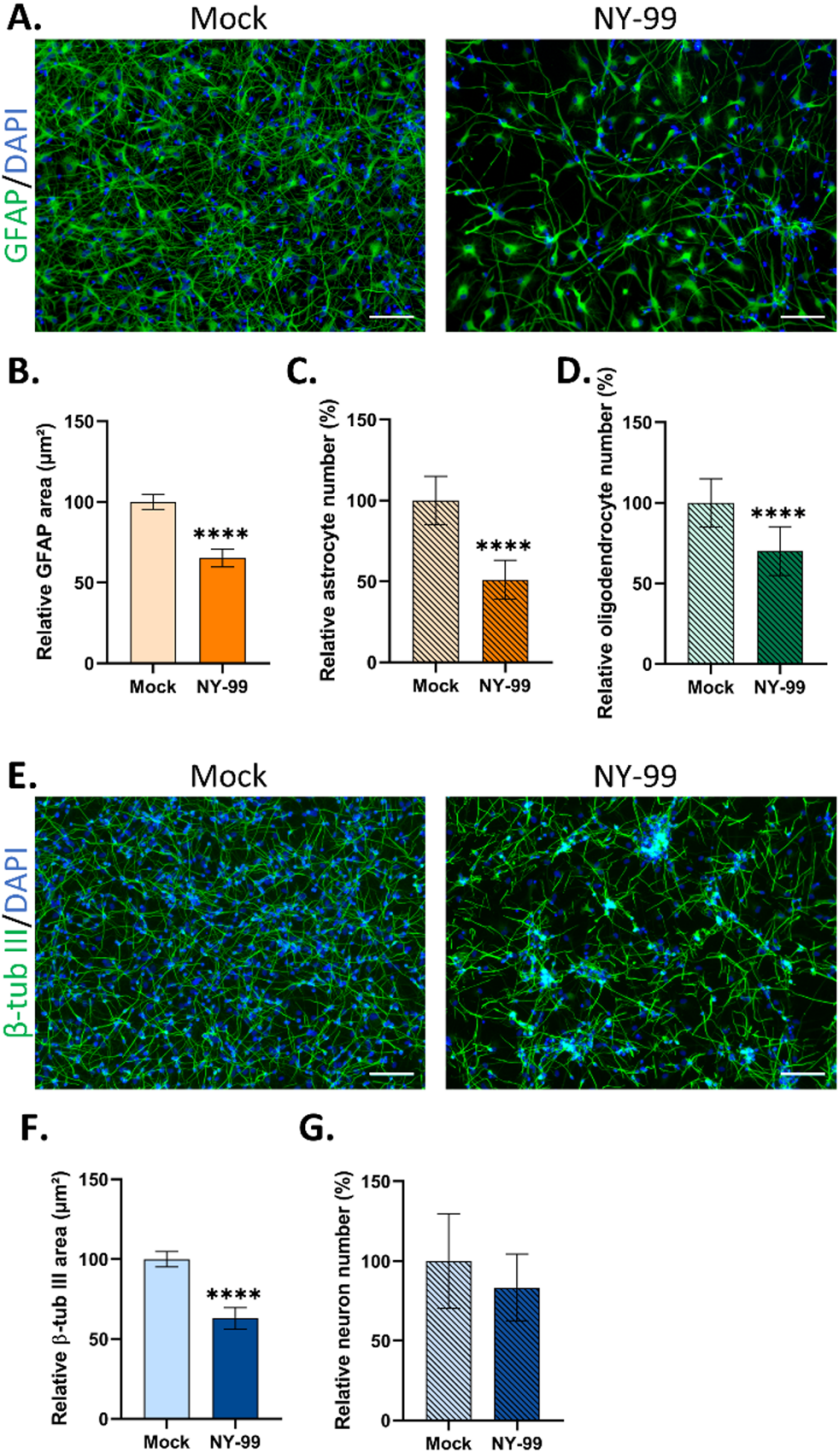
Cell damage induced by WNV_NY99_ in hNGC. Human NGC were infected with WNV_NY99_ at MOI 10 for 7 days. (**A**, **E**) Immunofluorescence labeling using antibodies against GFAP (astrocytes) (**A**) or βIII-tubulin (neurons) (**E**) in green. Nuclei were stained with DAPI (blue). Scale bars = 100 µm. (**B**, **F**) Automatic quantification of area occupied by astrocytes (**B**) and neurons (**F**) (cell bodies and processes), based on immunofluorescence staining using an ImageXpress micro instrument, (**C**) Automatic enumeration of astrocytes, based on nuclei characteristics, using an OPERA Phenix Plus instrument, (**D**, **G**) Automatic enumeration of Olig2-positive (oligodendrocytes) (**D**) and HuC/D-positive (neurons) (**G**) cells, using an ImageXpress micro instrument. The results are expressed as the mean ± SD and are representative of three independent experiments performed in 6 replicates (**C**) or are pooled from two independent experiments performed in at least 4 replicates (**B**, **D**, **F**, **G**). Results are normalized relative to uninfected cells (mock). Statistical analysis was performed using a two-tailed unpaired t test with GraphPad Prism V10.0.0. ***p < 0.001; ****p < 0.0001.

In order to evaluate whether WNV_NY99_ infection of astrocytes affects their morphology and survival, hNGC infected for 7 days were immunostained with an antibody directed against GFAP. Upon observation, a distinct pattern of GFAP labeling was detected in WNV-infected cells as compared with uninfected matched controls (Fig. 3A). In WNV_NY99_-infected cultures, immunostained cells presented large cell bodies with thick processes, reminiscent of astrogliosis. Upon quantification of the total surface area of GFAP staining, a diminution of 35% was observed in WNV_NY99_-infected hNGC (Fig. 3B). To determine whether the reduction was due to astrocyte death, we counted the number of astrocytes, revealing a loss of 49 ± 12% in this cell population (Fig. 3C). Oligodendrocytes, which were also infected by WNV_NY99_, were counted based on OLIG2 immunostaining. A 30% reduction in OLIG2-positive cells was observed in infected as compared with uninfected cultures (Fig. 3D), showing that infection also affected oligodendrocyte survival. Similar results were obtained for the WNV_Fr2015_ and WNV_Fr2018_ strains, showing no differences among strains in their capacity to damage glial cells in hNGC cultures (Supplemental Fig. 4A-D).

We next sought to determine whether neuronal cells, although uninfected, might be affected in their survival. We thus infected hNGC for 7 days and immunostained them with an antibody directed against βIII-tubulin. Microscopic observation revealed that neuronal cells formed clusters, suggesting possible neuronal stress (Fig. 3E). Quantification of the total area of βIII-tubulin labeling revealed a 37% decrease in WNV_NY99_-infected cells as compared with their matched uninfected controls (Fig. 3F). Once again, WNV_Fr2015_ and WNV_Fr2018_ strains behaved as did the WNV_NY99_ strain, though in a more marked manner, as they induced a 59% decrease in the total area exhibiting βIII-tubulin labeling (Supplemental Fig. 4E, F). Regarding loss of neurons, quantified on the basis of HuC/HuD immunostaining, although the diminution of 17% at 7 dpi in WNV_NY99_-infected cells did not reach statistical significance (Fig. 3G), significant decreases were evidenced in WNV_Fr2015_- and WNV_Fr2018_-infected cells (34% and 36%, respectively) (Supplemental Fig. 4G), demonstrating neuronal death. Thus, our results revealed that, despite strictly limited dissemination in human brain cell culture, WNV deeply impacted the survival of not only astrocytes and oligodendrocytes, but also uninfected neurons, resulting in death of all three cell types. These results show that WNV infection triggers both direct and indirect cell death.

**Fig. 4:**
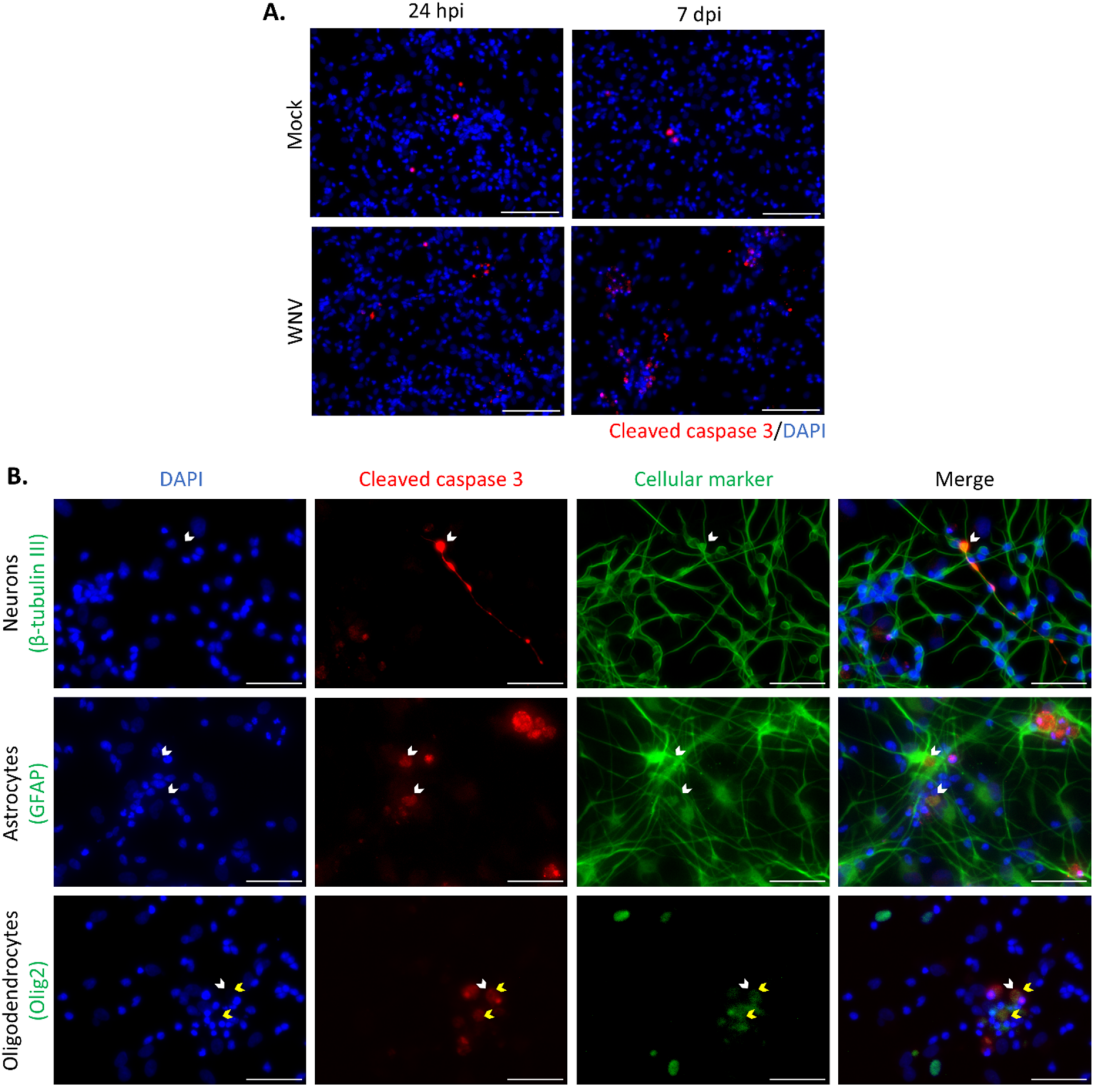
Apoptotic death in WNV-infected hNGC. Human NGC were infected with WNV_NY99_ at MOI 10. (**A**) Uninfected (mock) and infected (WNV) cells were immunostained with an antibody directed against cleaved caspase-3 (red) at 24 hpi and 7 dpi. Nuclei were stained with DAPI (blue). Scale bars = 100 µm, (**B**) Immunofluorescence labeling of infected cells at 7 dpi. Antibodies against βIII-tubulin (neurons), GFAP (astrocytes) or OLIG2 (oligodendrocytes) (green) and cleaved caspase 3 (red) were used. Note OLIG2 staining leakage outside the nucleus in apoptotic cells (yellow arrowheads). Nuclei were stained with DAPI (blue). White arrowheads show colocalization between cleaved caspase-3 and the different cellular markers. Scale bars = 50 µm.

To address the molecular mechanisms involved in cellular death, we infected hNGC for 24 h or 7 days and immunostained them with an antibody directed against cleaved-caspase 3 (C3A), a caspase that is central to apoptotic death. Observation of immunostained cells revealed a strong increase in the number of C3A-positive cells in WNV-infected hNGC as compared with uninfected hNGC at 7 dpi (Fig. 4A), thus showing that apoptotic death is involved in WNV-induced death. Immunostaining for C3A was observed in a proportion of cells that stained positive for either of the three cell type-specific markers—GFAP, βIII-tubulin or OLIG2 (Fig. 4B)—, indicating that apoptotic death occurred in all three cell types.

### WNV induces an inflammatory response in human neuronal/glial cells

**Fig. 5:**
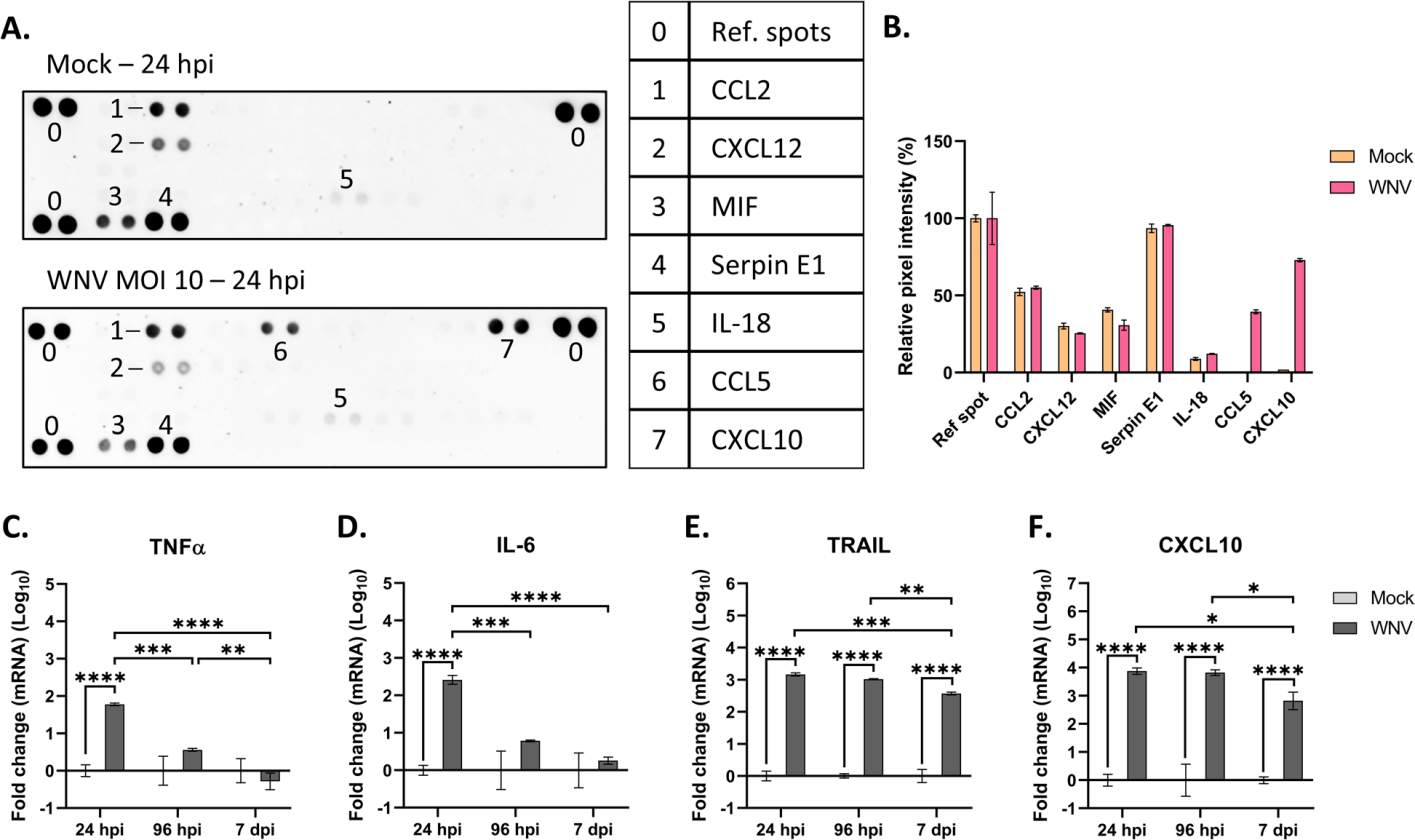
WNV-induced inflammatory response in hNGC. Human NGC were infected with WNV_NY99_ at MOI 10. (**A**) Immunoblot of 36 cytokines present in supernatant collected from infected (WNV) or uninfected (mock) cells at 24 hpi, using a Proteome Profiler Assay (R&D Systems), (**B**) Quantification of pixel intensity of each visible dot on the immunoblots. Results are expressed as the mean ± SD and represent one experiment performed in duplicate, (**C**–**F**) RT-qPCR analyses of selected cytokine genes. Gene expression was normalized to GADPH gene, and the − 2ΔΔCt method was used for relative quantification compared with uninfected cells (mock) at each timing. Data are expressed as the mean ± SD. Results are representative of two independent experiments performed in at least duplicate. Data were log-transformed (Y = log[Y]). Statistical analysis was performed using a two-tailed unpaired t test with GraphPad Prism V10.0.0. *p < 0.05; **p < 0.01; ***p < 0.001; ****p < 0.0001.

We have previously shown that neuronal/glial cells derived from human neural progenitors have the capacity to respond to tick-borne encephalitis virus (TBEV), another *Orthoflavivirus*, by producing an inflammatory response^20^. Here, we measured their response to WNV infection. Human NGC were infected with WNV_NY99_ at MOI 10 and the differential secretion of 36 pro-inflammatory cytokines and chemokines in the culture supernatant was evaluated at the peak of infection (24 hpi) using an immunoblotting approach. The studied proteins are shown in Supplemental table 1. Of the 36 proteins analyzed, 7 were secreted at levels sufficient for detection; namely, C–C motif chemokine ligand 2 (CCL2), CXC motif chemokine ligand 12 (CXCL12), macrophage inhibitory factor (MIF), serine protease inhibitor E1 (Serpin E1), interleukin 18 (IL-18), C–C motif chemokine ligand 5 (CCL5, also called RANTES (regulated upon activation, normal T-cell expressed and secreted) and CXC motif chemokine ligand 10 (CXCL10) (Fig. 5A). Among these, two factors, CCL5/RANTES and CXCL10, were differentially secreted, both showing a strong increase in WNV-infected hNGC supernatant in comparison with their matched uninfected controls (Fig. 5B). Tumor necrosis factor alpha (TNFα) and IL6, two neurotoxic cytokines that were previously shown to be upregulated by TBEV^20,29^, were not detected in the supernatants of WNV-infected hNGC with this approach, nor in the non-infected cultures. As the immunoblotting approach may not have been sufficiently sensitive, we further analyzed potential differential expression of the corresponding genes by RT-qPCR. The TNF-related apoptosis inducing ligand (TRAIL) gene, which encodes a neurotoxic cytokine that was not included in the immunoblot, was added to the analysis. At 24hpi, a 1.8-, 2.4- and 3.2-fold (log_10_) upregulation was observed for TNFα (Fig. 5C), IL6 (Fig. 5D) and TRAIL (Fig. 5E), respectively, showing that WNV indeed induced their expression. As a control, CXCL10 gene expression was also assessed and found to be strongly upregulated (3.9-fold (log_10_)) upon WNV infection (Fig. 5F), consistent with its increased secretion in hNGC supernatant (Fig. 5A, B). We next measured the kinetics of their expression from 24 hpi to 7 dpi. The upregulation of TNFα and IL6 was strongly reduced at 96 hpi, returning to baseline levels by 7 dpi. This reduction between 24 hpi and 7 dpi occurred in parallel with the decline in WNV infection over the same time frame (Fig. 1A–C). A decrease in gene expression was also observed for TRAIL and CXCL10, albeit later, at 7 dpi, and to a more modest degree, as their relative expression levels remained high (Fig. 5E, F). Thus, our results showed that WNV induced a pro-inflammatory response in hNGC, leading to the secretion of factors known to be capable of attracting T cells (CXCL10) and damaging neurons (TNFα, IL6, TRAIL and CXCL10).

### Type I IFN signaling blocks WNV dissemination in human astrocytes and oligodendrocytes but is not responsible for the lack of neuronal tropism

**Fig. 6:**
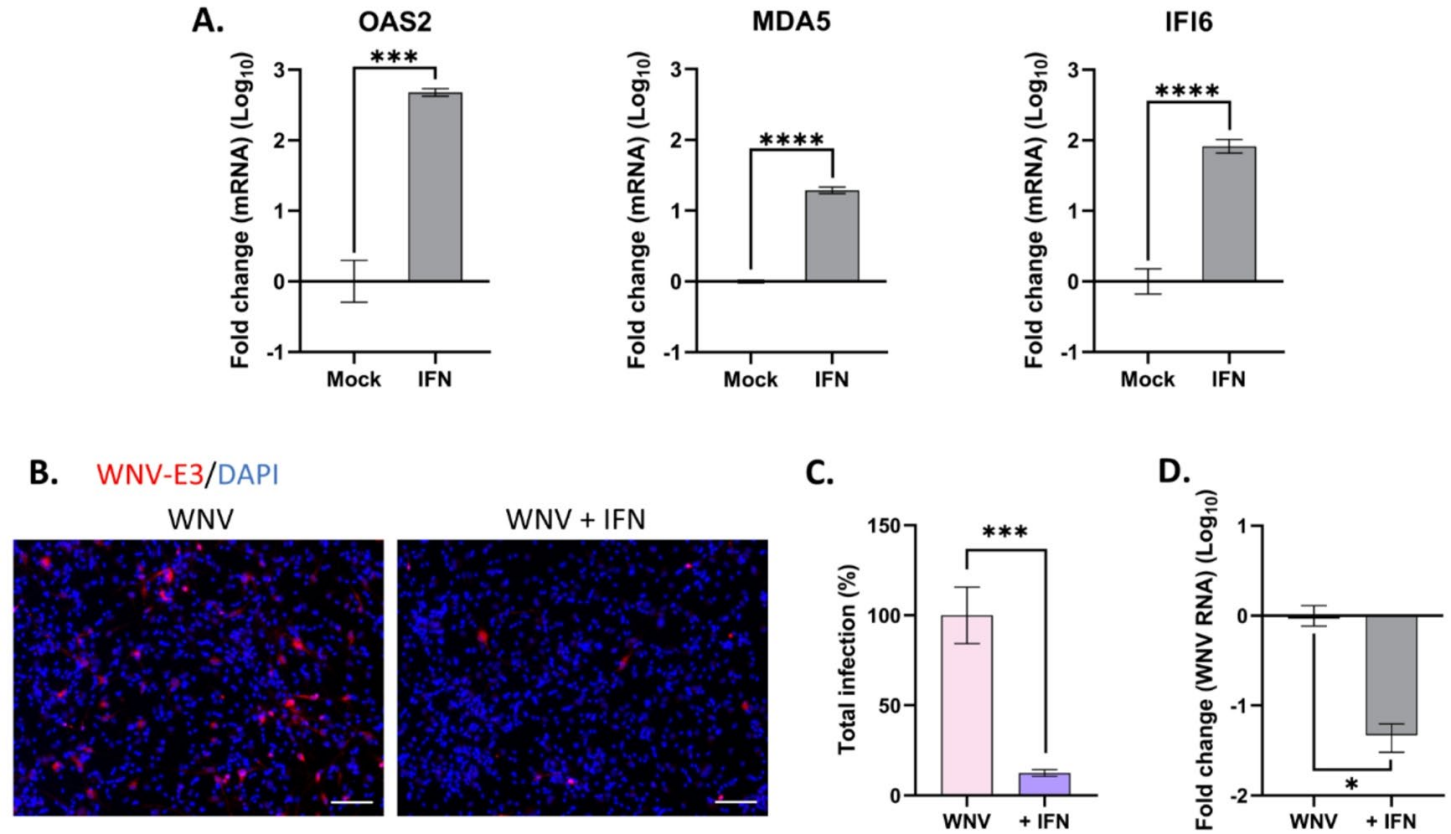
Exogenous IFN induced an antiviral response limiting WNV replication in hNGC. Human NGC were treated with IFN-β (100 U/mL) and/or infected with WNV at MOI 10 for 24 h. (**A**) RT-qPCR analyses of selected antiviral genes. Gene expression was normalized to the GADPH gene, and the − 2ΔΔCt method was used for relative quantification (compared with non-treated cells (mock)), (**B**) Immunofluorescence labeling with an antibody against WNV-E3 (red). Nuclei are stained with DAPI (blue). Scale bars = 100 µm, (**C**) Automatic enumeration of infected cells, based on immunofluorescence staining using an OPERA-Phenix™ Plus instrument, (**D**) RT-qPCR analyses of WNV genome in supernatant. Gene expression was compared with infected non-treated cells (WNV) for relative quantification. Data are expressed as the mean ± SD and were log-transformed (Y = log[Y]). Results are representative of two (**A**, **D**) or three (**C**) independent experiments performed in, at least, triplicate. Statistical analysis was performed using a two-tailed unpaired t test (**A**) or a two-tailed unpaired Mann–Whitney test (**C**, **D**) with GraphPad Prism V10.0.0. *p < 0.05; ***p < 0.001; ****p < 0.0001.

Type I IFN signaling is a potent inhibitor of flavivirus replication^30^. We previously showed that human neuronal/glial cells respond to TBEV infection by mounting a strong antiviral response through activation of the IFN signaling pathway^20^. Here, we sought to evaluate the role of IFN signaling in the control of WNV infection and dissemination in human astrocytes, oligodendrocytes and neurons. We first assessed the impact of exogenous IFN-β. One hundred units of IFN-β were added per milliliter for 24 h to 13-day old hNGC before quantification of the expression of three interferon-stimulated genes (ISGs) by RT-qPCR. 2’-5’-oligoadenylate synthetase 2 (OAS2), melanoma differentiation-associated protein 5 (MDA5) and interferon alpha inducible protein 6 (IFI6) were all upregulated, by 2.7-, 1.3- and 1.9-fold) (log_10_), respectively (Fig. 6A), confirming active IFN signaling in hNGC. The impact of IFN-β on WNV replication in hNGC was next assessed by pre-treating cells for 2 h (100 U/mL of IFN-β) before WNV infection (MOI 10) and quantifying infection by cell imaging and RT-qPCR at 24 hpi. IFN-β pretreatment led to a significant reduction in WNV infection compared with untreated controls (Fig. 6B), as confirmed by a striking 87% decrease in the number of infected cells (Fig. 6C). This inhibitory effect was further corroborated by a 1.3-log_10_ reduction in viral genomic RNA in IFN-β-treated cells compared to controls (Fig. 6D). These results showed that exogenous IFN-β effectively suppresses WNV replication in hNGC.

**Fig. 7:**
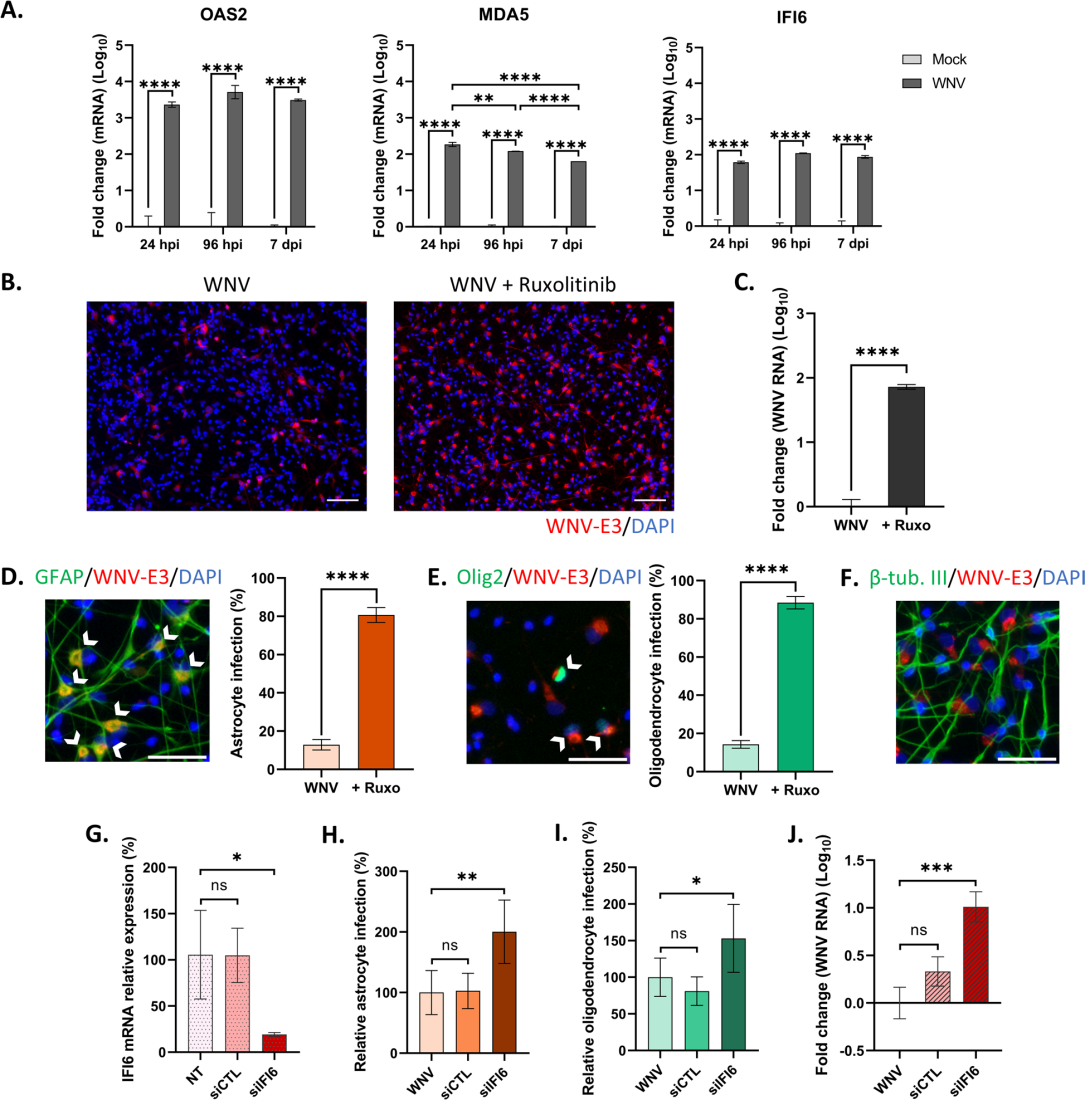
Endogenous IFN response controls WNV infection in astrocytes and oligodendrocytes but does not account for neuronal resistance to infection. (**A**) RT-qPCR analyses of selected antiviral genes from lysates of hNGC infected with WNV_NY99_ at MOI 10 for 24, 96 h or 7 days, (**B**–**F**) hNGC were pre-treated for 2 h with ruxolitinib (5 µM) before infection with WNV at MOI 10 for 24 h, (**B**) Immunofluorescence labeling with an antibody against WNV-E3 (red). Nuclei are stained with DAPI (blue). Scale bars = 100 µm, (**C**) RT-qPCR analyses of WNV genome in supernatant of infected cells, (**D**-**F**) Immunofluorescence labeling with antibodies directed against GFAP (**D**), OLIG2 (**E**) or βIII-tubulin (**F**) in green and WNV-E3 in red. Scale bars = 50 µm. Arrowheads show infected cells. Automatic enumeration of infected astrocytes (**D**) or oligodendrocytes (**E**), (**G**-**J**) hNGC were transfected with an siRNA targeting IFI6 mRNA or with a control (siCTL) (**G**), before infection with WNV (**H**–**J**), (**G**) Knockdown efficiency was assessed by RT-qPCR analysis. (**H**–**I**) Automatic enumeration of infected astrocytes (**H**) or oligodendrocytes (**I**), (**J**) RT-qPCR analyses of WNV genome in supernatant of infected cells. Automatic enumeration was based on immunofluorescence staining, using an OPERA-Phenix™ Plus instrument. For RT-qPCR analyses from lysates, gene expression was normalized to GADPH (**A**) or HPRT1 (**G**) genes, and the − 2ΔΔCt method was used for relative quantification, compared with uninfected cells (**A**) or non-transfected cells (**G**). For RT-qPCR analyses of WNV genome in supernatant, gene expression was normalized to infected untreated cells (**C**) or to infected non-transfected cells (**J**) for relative quantification. Data are expressed as the mean ± SD. Data displayed in **A**, **C** and **J** were log-transformed (Y = log[Y]). Statistical analysis was performed using a one-way ANOVA (**A**, **G**–**J**) or a two-tailed unpaired t test (**C**–**E**) with GraphPad Prism V10.0.0. ns = non-significant; *p < 0.05; **p < 0.01; ***p < 0.001; ****p < 0.0001.

We next wondered whether endogenous IFN signaling was involved in controlling WNV replication in hNGC. We observed that OAS2, MDA5 and IFI6 ISGs were all upregulated in WNV-infected hNGC from 24 hpi to 7 dpi (Fig. 7A), showing that WNV infection induced IFN signaling. Addition of ruxolitinib (5 µM), a strong inhibitor of the IFN signaling pathway, 2 h prior to WNV infection led to a dramatic increase in infection at 24 hpi, compared with untreated hNGC (Fig. 7B). This was confirmed by quantification of viral RNA in the supernatant, which showed a 1.9-fold (log_10_) increase in ruxolitinib-treated cells (Fig. 7C). In order to determine which cell types were affected, cells were examined after co-immunostaining with antibodies directed against a cellular marker (GFAP, OLIG2 or βIII-tubulin) and the E3 domain of the WNV envelope protein. The percentage of infected GFAP-positive cells and OLIG2-positive cells were both dramatically increased, rising to 80.7 ± 3.9% **(**Fig. 7D) and 88.5 ± 3.3% (Fig. 7E), respectively, demonstrating that the IFN signaling pathway played a major role in controlling WNV replication in these two cell types. In contrast, no βIII-tubulin-positive cells were co-immunostained with WNV antibody (Fig. 7F). Thus, WNV failed to infect neurons even when the IFN response was inhibited, suggesting that the absence of infection in neurons could not be attributed to induction of a protective IFN response in these cells. Finally, in order to gain insight into which ISG is involved in blocking WNV replication in human glial cells, we used an siRNA approach to knockdown IFI6 gene expression. To ascertain efficient knockdown, IFI6 transcripts were quantified by RT-qPCR 48 h after transfection, revealing an 80% downregulation in mRNA expression (Fig. 7G). Transfected hNGC were infected at this time point with WNV (MOI 10) for an additional 24 h, when the impact of IFI6 gene knockdown on WNV replication was assessed by cell imaging and quantification of genomic viral RNA in supernatant. Reduced IFI6 expression induced 2.0-fold and 1.5-fold increases in the percentage of WNV-infected astrocytes and oligodendrocytes, respectively, as compared with non-transfected hNGC (Fig. 7H, I), and a 1.0-fold (log_10_) increase in genomic viral RNA present in supernatants (Fig. 7J), showing that IFI6 contributed to the antiviral state against WNV in human glial cells.

## Discussion

WNV is a significant global health problem. While much progress in understanding its neuropathology has been achieved, our knowledge of the mechanisms involved in the human brain is still limited due to the lack of species-specific and physiologically relevant in vitro models. In this study, we addressed this gap. Using neuronal/glial cells derived from human fetal neural progenitors, we established a novel model of WNV infection and studied the relationship between tropism, innate and inflammatory responses and cellular damage. We observed that viral infection was cell type-specific, with glial cells—both astrocytes and oligodendrocytes—being permissive, and neurons unexpectedly resistant. We showed that IFN signaling is critical for restricting WNV tropism in glial cells, whereas it does not contribute to viral control in neurons. We further showed that substantial damage occurred in all three cell types, infected and uninfected, involving both direct viral effects and indirect mechanisms, some of which possibly driven by inflammatory components, underscoring a complex interplay between neurons and glia.

Like other neurotropic flaviviruses, WNV preferentially targets neurons, as shown in rodent^31,32^ and human tissues^11–13^. In vitro studies using rodent^32–34^ or human cells^35–37^ support this observation, consistently reporting high neuronal permissiveness. However, neuronal subpopulations of the human brain are differentially permissive to WNV. Indeed, post-mortem analyses revealed infection in Purkinje cells, neurons of the spinal cord, substantia nigra, hippocampus, and entorhinal cortex, but not in cerebellar granule cells or neurons of the cingulate and insular cortex, despite local inflammation suggesting infection of these brain areas^11^. Similarly, WNV-infected neurons in rhesus macaques were restricted to motor control regions^38^, suggesting subtype-specific tropism. Here, we provide the first evidence that human neural cultures contain neurons refractory to WNV infection^35–37^. This was consistent across three viral strains (WNV_NY99_, WNV_Fr2015_, WNV_Fr2018_) from both lineages 1 and 2, and parallels findings in iPSC-derived equine neurons^39^, indicating that neuronal refractoriness is not species-restricted. We explored possible explanations for the resistance of human neurons to WNV in our cultures. While murine studies suggested that antiviral responses shape neuronal susceptibility^40^, this mechanism seems unlikely here: human neurons mount weaker IFN responses than astrocytes in our cultures – at least against TBEV^20^ –, and inhibiting IFN signaling with ruxolitinib did not restore permissiveness. Instead, refractoriness likely reflects the absence of essential host factors or the presence of restriction factors that are independent of IFN signaling. These permissive or restrictive factors are lost or gained, respectively, during neuronal differentiation, as fetal neural progenitor cells from which hNGC are derived are highly permissive to WNV^39^. They appear to be specific to WNV as TBEV, another orthoflavivirus, massively infects neurons in our cultures^20^. This may underlie the heterogeneity of neuronal infection patterns observed in human patients^11^. Our study therefore establishes the first human neural model reproducing neuronal refractoriness to WNV. It underscores the importance of using multiple in vitro models that altogether reflect with greater fidelity the complexity of WNV interactions with human neural tissue and allow the study of mechanisms of both viral- and immune-mediated neuronal injury. Although our results do not reveal which specific step(s) of the viral replication cycle are blocked, and determining them is beyond the scope of this study, further investigations using this model may provide valuable insights into the mechanisms governing WNV replication in neurons.

Unlike neurons, human astrocytes and oligodendrocytes supported WNV replication in our cultures. While astrocyte infection has been reported in rodents^14^ and humans^13^, evidence for oligodendrocyte infection in vivo is lacking. To our knowledge this is the first demonstration of WNV replication in primary-like oligodendrocytes, beyond immortalized cell lines^41^. This warrants further examination of human brain tissues as their infection in vivo may have been previously overlooked. In both glial cell types, viral replication peaked at 24 h and declined by 48 h, consistent with effective antiviral control. In astrocytes, restriction of WNV by IFN signaling is well established in murine models^42^, but oligodendrocyte responses remained unexplored. Oligodendrocytes are, however, known to be capable of responding to IFN, although in a less robust manner than microglia^43^. Here, we show that WNV-infected glial cells upregulate IFN-stimulated genes, and that both endogenous and exogenous IFNs strongly limit WNV replication in human astrocytes and oligodendrocytes. These findings highlight the central role of IFN signaling in glial restriction of WNV. They extend previous findings observed in rodent’s astrocytes and provide the first evidence for this mechanism in human oligodendrocytes.

Recent studies have identified several ISGs with antiviral activity against WNV (reviewed in^44^). Among these, IFI6 was characterized as one of the most important IFN-inducible effectors against orthoflaviviruses^45^. Its functional role in human neuronal and glial cells, however, had not yet been addressed. Our results showed that downregulation of IFI6 in WNV-infected hNGC cultures led to a significant increase in the number of infected cells, providing the first evidence that it contributes to the control of WNV replication in human glial cells. Whether IFI6 plays a predominant role in this context remains to be confirmed and will require future studies using complete gene knockout approaches.

Contrary to our expectation that rapid control of WNV infection in glial cells would preserve cellular integrity, we observed extensive pathological effects across all three cell types (astrocytes, oligodendrocytes and neurons) by 7 dpi, in both infected and uninfected cells. Thus, even when viral dissemination is suppressed, WNV can severely impact neural cells through both direct and indirect mechanisms.

At 7 dpi, astrocytes exhibited both reactivity and death. While astrocyte activation during WNV infection is well established^13,46^, it is generally believed that persistent infection occurs without overt death, as shown in isolated primary astrocytes^34^. Infection in murine organotypic cultures^47^ and human mixed neural cultures^37^ have however reported significant loss. These conflicting observations may reflect astrocyte heterogeneity^48^ or the influence of cell interactions present in complex culture systems but absent in isolated models. In our hNGC cultures, astrocyte death (49–57%, depending on WNV strain) far exceeded the proportion of infected cells (~ 12% at peak), indicating contributions from both direct viral cytotoxicity and indirect mechanisms. Together with previous reports, these findings thus underscore that cellular context critically determines astrocyte outcomes in neurotropic infections.

We also observed infection and subsequent death of oligodendrocytes in hNGC cultures. Orthoflaviviruses differ in their effects on oligodendrocytes. While ZIKV induces apoptotic death^49^, TBEV infection occurs without evident damage^20^. To our knowledge, this is the first demonstration that WNV both infects and harms oligodendrocytes. As with astrocytes, the proportion of dying cells (24–48%) exceeded the percentage of infected cells (~ 18% at peak), suggesting again contributions from both direct cytotoxicity and indirect, inflammatory mechanisms. This interpretation is supported by the sensitivity of oligodendrocytes to cytokines such as TNFα^50^ and CXCL10^51^, both being upregulated for several days in our cultures. The significance of oligodendrocyte infection and death during WNV neuroinvasion remains uncertain. Demyelination is rarely reported in human cases, arguing against a major loss of mature oligodendrocytes. However, murine studies suggest that oligodendrocyte death can occur, resulting in the release of the IL-33 cytokine, an alarmin which plays a significant role in microglial activation and consecutive brain inflammation^52^. Our findings thus call for further investigation into the contribution of oligodendrocytes to WNV neuropathogenesis.

Although neurons were refractory to WNV infection in our cultures, they nonetheless sustained substantial damage, as shown by cell surface reduction across the three viral strains and by neuronal death in WNV_Fr2015_- and WNV_Fr2018_-infected cultures. Neuronal injury is a hallmark of neurotropic orthoflavivirus infections. It has been largely attributed to direct viral infection and subsequent apoptosis^33,53,54^. Nevertheless, a role for indirect mechanisms — also called bystander effects — in damage of uninfected cells has been evidenced (reviewed in^53^). Bystander effects are notably attributed to the release of pro-inflammatory factors by infected or activated glial cells^13,55^. Our study provides the first direct demonstration that WNV-infected astrocytes and oligodendrocytes can mediate significant neuronal damage in a physiologically relevant human model. This damage coincided with upregulation of inflammatory mediators such as IL6, TNFα, TRAIL, and CXCL10. Of note, the relative contribution of astrocytes and oligodendrocytes in upregulating pro-inflammatory factors cannot be fully established in our cultures, as both cellular types can produce these factors^20,56^. However, they are most likely produced by astrocytes given their relative abundance. Thus, WNV infection of glia is sufficient to harm uninfected neurons, extending previous work in transformed cell lines^13^. Additional mechanisms may also contribute, including impaired trophic support from reactive astrocytes^57^, a phenomenon which may be amplified by the reduction in the number of astrocytes or by other mechanisms such as pathological accumulation of amyloid-β, as recently suggested^58^.

Notably, hNPC-derived hNGC cultures do not contain microglia, which are key players in the CNS response to WNV infection^59^. Whether their presence would modify the impact of WNV on neurons, astrocytes and oligodendrocytes remains to be determined.

In conclusion, our study provides the first comprehensive analysis of the relation between WNV tropism, innate and inflammatory responses, and cell damage in human neuronal/glial cultures. It both confirmed previous findings obtained in murine models or less physiologically relevant in vitro models and revealed novel cellular and molecular mechanisms that may be involved in WNV-induced neuropathology in the human brain. Finally, it provides a novel in vitro model to further question the mechanisms of neuropathology and assess new therapeutics.

## Supplementary Information

Below is the link to the electronic supplementary material.

Supplementary Material 1

## Data Availability

All data generated or analysed during this study are included in this published article (and its Supplementary Information files).

## Abbreviations

bFGF: Basic fibroblast growth factor
CCL2: C–C motif chemokine ligand 2
CCL5: C–C motif chemokine ligand 5
CNS: Central nervous system
CXCL10: CXC motif chemokine ligand 10
CXCL12: CXC motif chemokine ligand 12
dsRNA: Double-stranded RNA
EGF: Epidermal growth factor
GFAP: Glial fibrillary acidic protein
hNGC: Human neuronal/glial cells
hNPC: Human neural progenitor cells
IFI6: Interferon alpha inducible protein 6
IFN: Interferon
IL: Interleukin
MDA5: Melanoma differentiation-associated protein 5
MIF: Macrophage inhibitory factor
OAS2: 2’-5’-Oligoadenylate synthetase 2
OLIG2: Oligodendrocyte transcription factor 2
RANTES: Regulated upon activation: normal T-cell expressed and secreted
RT-qPCR: Reverse transcriptase quantitative polymerase chain reaction
Serpin E1: Serine protease inhibitor E1
TNFα: Tumor necrosis factor alpha
TRAIL: TNF-related apoptosis inducing ligand
WNV: West Nile virus
