# Supplementary material for: Role of innate immune and inflammatory signaling in West Nile virus tropism and neuronal and glial cell death
Source: Sci Rep. 2025 Dec 19;15:44163. doi: 10.1038/s41598-025-27954-2 (PMC12717255; doi:10.1038/s41598-025-27954-2)
Supplement: Supplementary file 1 — Supplementary Material 1 [file 41598_2025_27954_MOESM1_ESM.docx]

**Supplemental figure 1.**


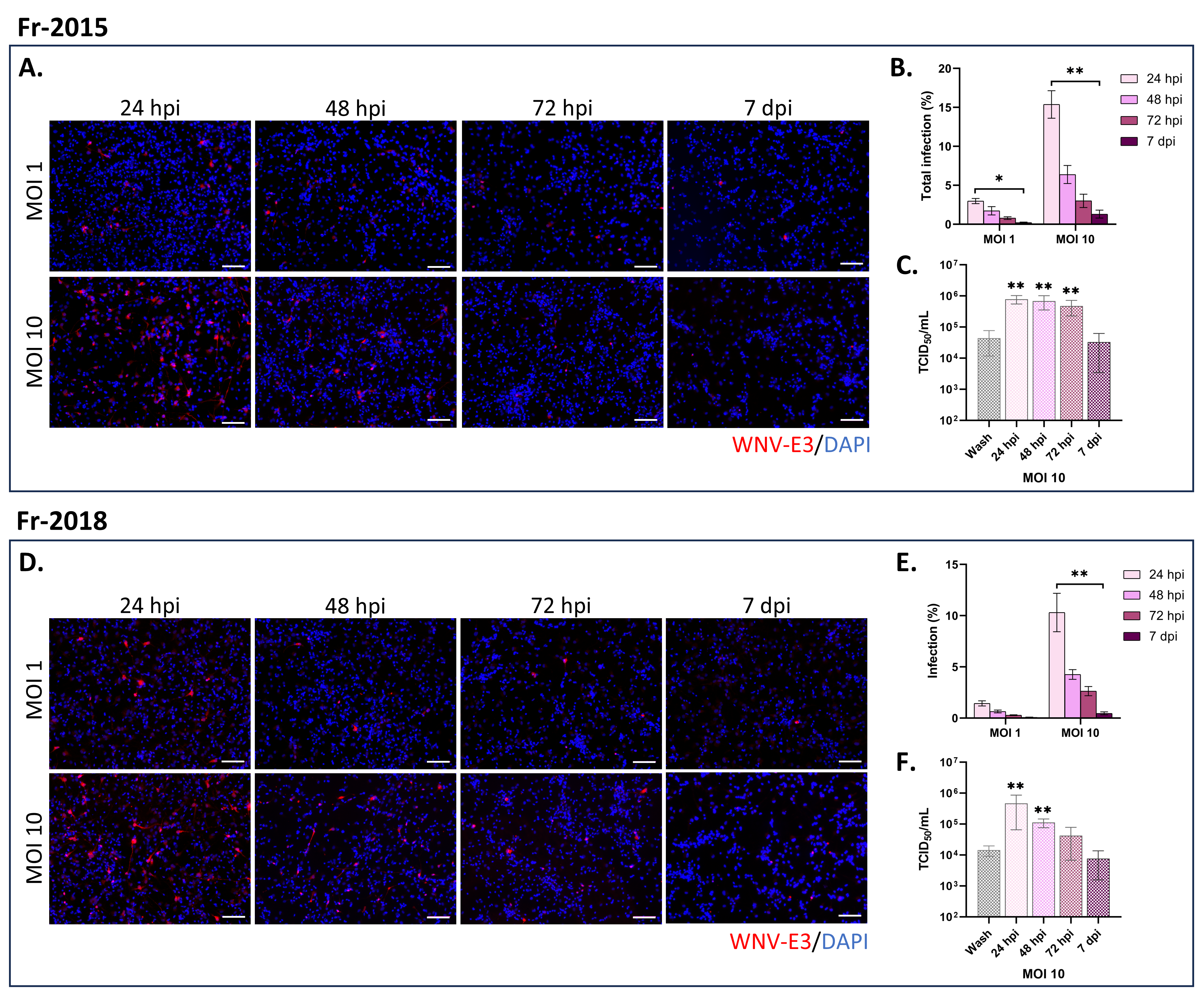


**Permissivity of neuronal/glial cells derived from human neural progenitors to WNV_Fr2015_ and WNV_Fr2018_**. (A, D) Immunofluorescence labeling with an antibody against WNV-E3 (red) of hNGC infected for 24, 48, 72 hours or 7 days with WNV_Fr2015_ (A) or WNV_Fr2018_ (D) at MOI 1 or 10. Nuclei are stained with DAPI (blue). Scale bars = 100 µm. (B, E) Automatic enumeration of cells infected with WNV_Fr2015_ (B) or WNV_Fr2018_ (E) based on immunofluorescence staining using an OPERA-Phenix™ Plus instrument. (C, F) Supernatant of hNGC infected with WNV_Fr2015_ (C) or WNV_Fr2018_ (F) at MOI 10 were titrated by endpoint dilution (TCID50) at the indicated time points. Results are representative of three independent experiments performed in six replicates (B, E) or pooled from two independent experiments performed in duplicate or triplicate (C, F). Data are expressed as the mean ± SD. Statistical analysis was performed using a Kruskal-Wallis test with post-hoc Dunn’s test (B, E) or two-tailed unpaired Mann-Whitney tests between the wash and other timepoints (C, F) with GraphPad Prism V10.0.0. *p < 0.05; **p < 0.01.

**Supplemental figure 2.**


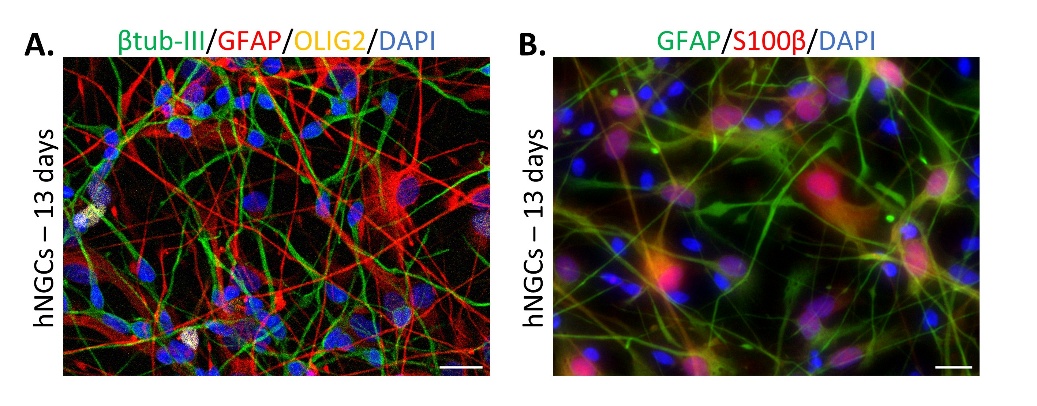


**Characterization of neuronal/glial cells derived from human neural progenitors.** (A, B) Immunofluorescence labeling of hNGC differentiated for 13 days. (A) Cells labeled with antibodies against βIII-tubulin (neurons, green), GFAP (astrocytes, red) and OLIG2 (oligodendrocytes, yellow). (B) Cells labeled with antibodies against GFAP (green) and S100β (red). Nuclei were stained with DAPI (blue). Scales bars = 20 µm.

**Supplemental figure 3.**


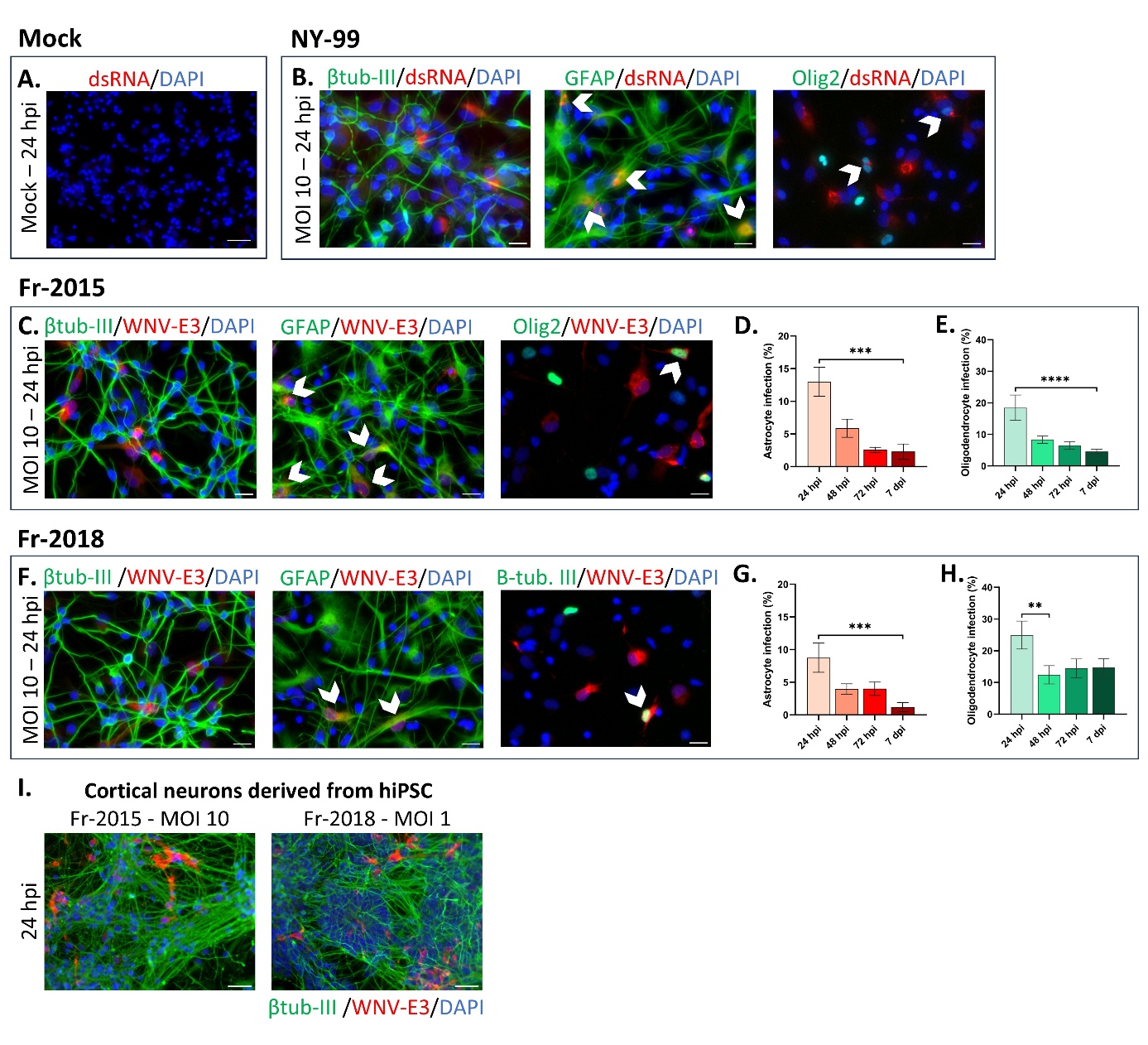
**WNV_NY-99_, WNV_Fr2015_ and WNV_Fr2018_ tropism in hNGC and cortical neurons derived from hiPSC.** Human NGC were infected with WNV_NY99_, WNV_Fr2015_ or WNV_Fr2018_ at MOI 10 for 24 hours. (A-B) Immunofluorescence labeling of dsRNA. (A) Mock-cells, (B) WNV_NY-99_-infected cells. Antibodies against βIII-tubulin (neurons), GFAP (astrocytes) or OLIG2 (oligodendrocytes) (green) and dsRNA (red) were used. (C, F) WNV_FR2015_- and WNV_Fr2018_-infected cells. Antibodies against βIII-tubulin (neurons), GFAP (astrocytes) or OLIG2 (oligodendrocytes) (green) and WNV-E3 (red) were used. Scale bars = 20 µm. (D, E, G, H) Automated enumeration of infected astrocytes (D, G) or oligodendrocytes (E, H) at 24, 48, 72 hpi and 7 dpi, based on immunofluorescence staining using an OPERA-Phenix™ Plus instrument. (I) hiPSC-derived cortical neurons were infected with WNV_Fr2015_ and WNV_Fr2018_ for 24 hours and labeled with antibodies against βIII-tubulin (green) and WNV-E3 (red). Scale bars = 50 µm. (B, C, F, I) Note that βIII-tubulin positive cells were not infected. Arrowheads show infected astrocytes and oligodendrocytes. Nuclei were stained with DAPI (blue). Results are representative of three independent experiments performed in at least 5 replicates. Data are expressed as mean ± SD. Statistical analysis was performed using a Kruskal-Wallis test with post-hoc Dunn’s test with GraphPad Prism V10.0.0. **p < 0.01; ***p < 0.001; ****p < 0.0001.

**Supplemental figure 4.**


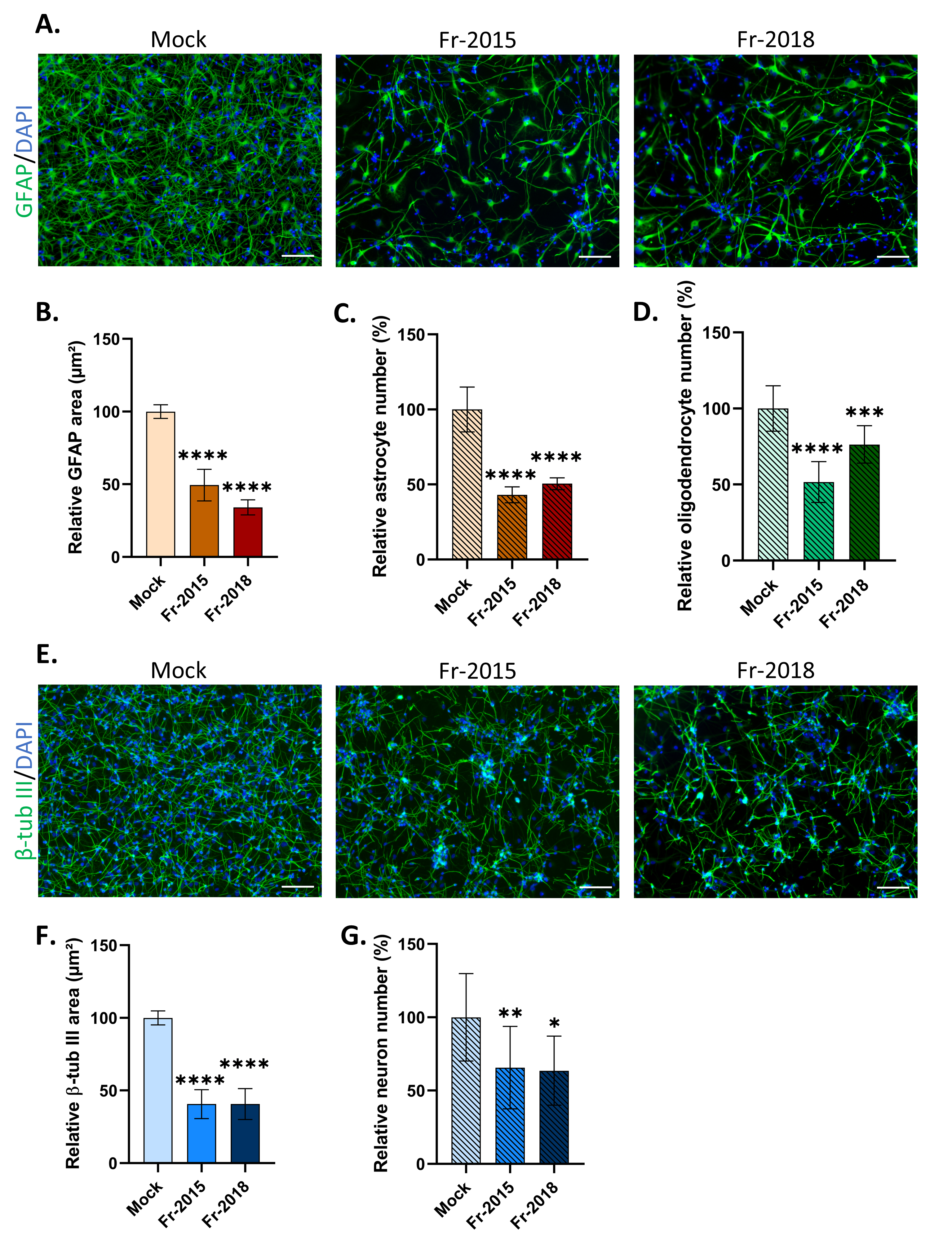


**Cell damage induced by WNV_Fr2015_ and WNV_Fr2018_ in hNGC.** Human NGC were infected with WNV_Fr2015_ or WNV_Fr2018_ at MOI 10 for 7 days. (A, E) Immunofluorescence labeling using antibodies against GFAP (astrocytes) (A) or βIII-tubulin (neurons) (E) in green. Nuclei were stained with DAPI (blue). Scale bars = 100 µm. (B, F) Automatic quantification of area occupied by astrocytes (B) and neurons (F) (cell bodies and processes), based on immunofluorescence staining using an ImageXpress micro instrument. (C) Automatic enumeration of astrocytes, based on nuclei characteristics, using an OPERA Phenix Plus instrument. (D, G) Automatic enumeration of Olig2-positive (oligodendrocytes) (D) and HuC/D-positive (neurons) (G) cells, using an ImageXpress micro instrument. The results are expressed as the mean ± SD and are representative of three independent experiments performed in 6 replicates (C) or are from a single experiment performed in 6 replicates (WNV_Fr2018_) or pooled from two independent experiments performed in 6 replicates (WNV_Fr2015_) (B, D, F, G). Results are normalized relative to mock cells. Statistical analysis was performed using a two-tailed unpaired t test with Graphpad Prism V10.0.0. ***p < 0.001; ****p < 0.0001.

**Supplemental table 1. List of cytokines tested in the Proteome Profiler Human Cytokine Array (R&D Sciences) and their coordinates on the blot.** Lines are named A to E, from top to bottom of the blot. Dots are numbered 1 to 20, from left to right.

| Coordinates | Gene name |
| --- | --- |
| A1, A2 | **Reference Spots** |
| A3, A4 | **CCL1/I-309** |
| A5, A6 | **CCL2/MCP-1** |
| A7, A8 | **MIP-1α/MIP-1β** |
| A9, A10 | **CCL5/RANTES** |
| A11, A12 | **CD40 Ligand/TNFSF5** |
| A13, A14 | **Complement Component C5/C5a** |
| A15, A16 | **CXCL1/GROα** |
| A17, A18 | **CXCL10/IP-10** |
| A19, A20 | **Reference Spots** |
| B3, B4 | **CXCL11/I-TAC** |
| B5, B6 | **CXCL12/SDF-1** |
| B7, B8 | **G-CSF** |
| B9, B10 | **GM-CSF** |
| B11, B12 | **ICAM-1/CD54** |
| B13, B14 | **IFN-γ** |
| B15, B16 | **IL-1α/IL-1F1** |
| B17, B18 | **IL-1β/IL-1F2** |
| C3, C4 | **IL-1ra/IL-1F3** |
| C5, C6 | **IL-2** |
| C7, C8 | **IL-4** |
| C9, C10 | **IL-5** |
| C11, C12 | **IL-6** |
| C13, C14 | **IL-8** |
| C15, C16 | **IL-10** |
| C17, C18 | **IL-12 p70** |
| D3, D4 | **IL-13** |
| D5, D6 | **IL-16** |
| D7, D8 | **IL-17A** |
| D9, D10 | **IL-17E** |
| D11, D12 | **IL-18/IL-1F4** |
| D13, D14 | **IL-21** |
| D15, D16 | **IL-27** |
| D17, D18 | **IL-32α** |
| E1, E2 | **Reference Spots** |
| E3, E4 | **MIF** |
| E5, E6 | **Serpin E1/PAI-1** |
| E7, E8 | **TNF-α** |
| E9, E10 | **TREM-1** |
| E19, E20 | **Negative Control** |

**Supplemental table 2.** Primer pairs used for RT-qPCR analyses.

| Gene name | Forward primer | Reverse primer |
| --- | --- | --- |
| CXCL10 | GCAGGTACAGCGTACGGTTC | CAGCAGAGGAACCTCCAGTC |
| GAPDH | CACCATCTTCCAGGAGCGAG | GAGATGATGACCCTTTTGGC |
| HPRT1 | GGACTAATTATGGACAGGACT | GCTCTTCAGTCTGATAAAATCTAC |
| IFI6 | TCGCTGATGAGCTGGTCTGC | ATTACCTATGACGACGCTGC |
| IL-6 | FCCTTCTCCACAAGCGCCTTC | AAGGCAGCAGGCAACACCA |
| MDA5 | TGCCCATGTTGCTGTTATGT | GTCTGGGGCATGGAGAATAA |
| OAS2 | TGTTTTCCGTCCATAGGAGC | CTGATCGACGAGATGGTGAA |
| TNFα | AGATGATCTGACTGCCTGGG | TGCTTGTTCCTCAGCCTCTT |
| TRAIL | AGCAATGCCACTTTTGGAGT | TTCACAGTGCTCCTGCAGTC |
| WNV | CCTGTGTGAGCTGACAAACTTAGT | GCGTTTTAGCATATTGACAGCC |
